# Per- and Polyfluoroalkyl Substances (PFAS) in Wild Boar (*Sus scrofa*): A Review of Toxicokinetics, Environmental Biomonitoring, and Human Exposure

**DOI:** 10.3390/toxics14100867

**Published:** 2026-09-29

**Authors:** Jelena Kaurinović, Ines Varga, Nina Bilandžić, Bruno Čalopek, Maja Đokić, Ivana Varenina, Božica Solomun Kolanović, Marija Sedak, Ivica Bošković, Elena Fattore

**Affiliations:** 1 Laboratory for Residue Control, Department of Veterinary Public Health, Croatian Veterinary Institute, Savska cesta 143, 10000 Zagreb, Croatia; kaurinovic@veinst.hr (J.K.); varga@veinst.hr (I.V.); calopek@veinst.hr (B.Č.); dokic@veinst.hr (M.Đ.); kurtes@veinst.hr (I.V.); solomun@veinst.hr (B.S.K.); sedak@veinst.hr (M.S.); 2 Department of Animal Production and Biotechnology, Faculty of Agrobiotechnical Sciences, Josip Juraj Strossmayer University of Osijek, 31000 Osijek, Croatia; ivica.boskovic@fazos.hr; 3 Centre of Preclinical Toxicology and Biochemistry, Istituto di Ricerche Farmacologiche Mario Negri IRCCS, 20156 Milano, Italy; elena.fattore@marionegri.it

**Keywords:** PFAS, wild boar, wildlife, tissue distribution, food safety, dietary exposure

## Abstract

Per- and polyfluoroalkyl substances (PFAS) are a large group of highly persistent synthetic chemicals widely distributed in the environment and transfer through food webs. Among wildlife species, wild boar (*Sus scrofa*) has emerged as a particularly relevant terrestrial bioindicator because of its omnivorous feeding ecology, rooting behavior, and continuous exposure to heterogeneous environmental compartments. Available evidence indicates that PFAS distribution in wild boar is strongly influenced by compound-specific physicochemical properties and biological factors. Due to their affinity for proteins, PFAS predominantly accumulate in protein-rich tissues, particularly liver and kidney, while considerably lower concentrations are generally observed in muscle tissue. Long-chain compounds, especially PFOS, are consistently reported as dominant contaminants. PFAS concentrations in wild boar vary substantially according to habitat characteristics. Studies have demonstrated clear associations between contamination levels in wild boar tissues and environmental matrices such as soil, as well as links with land use and industrial activity, supporting the value of wild boar as a sensitive bioindicator of terrestrial PFAS contamination. From a food safety perspective, dietary PFAS exposure varies substantially depending on the tissue consumed and consumption frequency; while occasional consumption of muscle tissue generally contributes minimally to overall dietary intake, high consumption rates—particularly by hunters and their families—or consumption of offal, such as liver, can represent a significant exposure pathway.

## 1. Introduction

Per- and polyfluoroalkyl substances (PFAS) constitute a large and structurally diverse group of synthetic organic chemicals that do not occur naturally. They have been produced since the 1940s, and it is currently estimated that more than 15,000 different compounds exist [1,2]. Their defining characteristic is the presence of carbon–fluorine bonds, formed through the partial (polyfluoro-) or complete (perfluoro-) substitution of hydrogen atoms with fluorine. These bonds are among the strongest in organic chemistry, conferring exceptional thermal stability, resistance to chemical degradation, and pronounced water- and oil-repellent properties [3,4,5].

As a result of these unique physicochemical properties, PFAS have been widely applied in numerous industrial processes and consumer products. Their use includes applications in firefighting foams, refrigeration systems, surface treatments for textiles, leather, and paper to enhance resistance to water, grease, and dirt. They are also present in food contact materials, non-stick coatings, personal care products, and various construction materials. This extensive use has resulted in multiple pathways through which PFAS are released into the environment [6,7,8].

Nevertheless, when considered from an environmental perspective, these same properties that make PFAS highly useful also give rise to significant concern. Due to their chemical stability and resistance to degradation, PFAS persist in environmental compartments such as soil, water, and air, where they can remain for extended periods. However, the persistence of PFAS varies considerably among individual compounds, as reflected by differences in their dissipation half-lives (DT50; time required for 50% of a substance to disappear). For example, reported DT50 values for PFAS precursors include 14 days for PFOAAm, 266–630 days for PFOAB, and 675 days for PFOSB, demonstrating substantial differences in persistence among individual compounds. Importantly, transformation of PFAS precursors can generate terminal perfluoroalkyl acids, such as PFOA and PFOS, which are highly resistant to further degradation with estimated half-lives exceeding decades [9,10,11,12,13]. Their ability to undergo long-range transport further facilitates their widespread distribution across different regions. Both atmospheric and oceanic pathways contribute to the transport of PFAS to remote environments. For example, PFAS have been detected in Arctic ice cores, with PFCA deposition fluxes reaching up to 8200 ng/m^2^/year, providing evidence of long-range atmospheric transport to remote regions [14]. Oceanic transport also contributes to their redistribution, with PFAS mass flows of 112 mg/year northward and 100 mg/year southward across the Fram Strait [15].

Beyond environmental persistence, many PFAS compounds also exhibit bioaccumulative behavior, allowing them to accumulate in biological tissues and transfer through food webs. Consequently, PFAS have been detected in a wide range of organisms and ecosystems globally [16,17].

Due to their widespread occurrence and persistence, increasing concern has been raised as growing evidence of adverse effects of PFAS on both human and animal health.

Exposure to certain PFAS has been associated with effects across multiple physiological systems, including disruptions in lipid metabolism, immune and endocrine function, as well as reproductive and developmental processes. Additionally, toxic effects on the liver and kidneys and an increased risk of certain cancers have been reported for specific compounds within this group [1,18,19]. Of the adverse effects listed, the European Food Safety Authority (EFSA) identified immune system effects, particularly reduced response to vaccination, as the most critical health endpoint associated with PFAS exposure [13]. Subsequent evidence has further strengthened concerns regarding carcinogenic potential, with the International Agency for Research on Cancer (IARC) classifying perfluorooctanoic acid (PFOA) as carcinogenic to humans (Group 1) and perfluorooctane sulfonate (PFOS) as possibly carcinogenic to humans (Group 2B) in 2025 [20].

PFOS and PFOA are among the most extensively studied PFAS and have been restricted or banned in many regions worldwide including through their listing under the Stockholm convention on persistent organic pollutants [21,22]. In the European Union, regulatory control of PFAS in food is currently addressed under Commission Regulation (EU) 2023/915, which sets maximum levels for four PFAS compounds—PFOA, PFOS, perfluorononanoic acid (PFNA), and perfluorohexane sulfonate (PFHxS)—as well as for their combined sum [23]. In parallel, restrictions on the manufacture, placing on the market, and use of specific PFAS have been established under the EU REACH framework, including restrictions on C9–C14 perfluorocarboxylic acids and, more recently, perfluorohexanoic acid (PFHxA) and related substances [24,25,26]. The increasing regulatory restrictions on individual PFAS have contributed to the use of alternative PFAS compounds with similar functional properties, including 4,8-dioxa-3H-perfluorononanoic acid (DONA), hexafluoropropylene oxide dimer acid (HFPO-DA, also known as GenX), and 6:2 chlorinated polyfluorinated ether sulfonate (6:2 Cl-PFESA) [2,27,28]. In response to this pattern, the European Union regulatory strategies are increasingly shifting toward a group-based approach, aiming to regulate PFAS collectively and thereby prevent the substitution of well-characterized hazardous substances with less studied, yet potentially equally harmful, alternatives [29].

Within the context of widespread environmental contamination by PFAS and their transfer through ecosystems, wild boar (*Sus scrofa*) represents a relevant link between environmental contamination, wildlife exposure, and potential human exposure. Due to their free-ranging nature and continuous interaction with terrestrial environments, wild boars may integrate PFAS exposure from multiple environmental compartments and dietary sources [30]. As wild boar meat and offal are also consumed by humans, particularly in hunting contexts, the species is of relevance not only for environmental monitoring but also for food safety. However, the interpretation of PFAS concentrations in wild boar cannot rely solely on their detection in individual tissues. The relationship between external environmental contamination and measured tissue concentrations is influenced by compound-specific toxicokinetics, biological retention, tissue distribution, and ecological and individual factors affecting exposure [31,32,33].

This narrative review synthesizes current knowledge on PFAS in wild boar, including their toxicokinetics, tissue distribution, suitability as a bioindicator of environmental contamination, and their relevance for human exposure through consumption. Rather than considering these aspects separately, the review highlights their interconnections, showing how environmental contamination influences exposure, while compound-specific toxicokinetics and tissue distribution determine PFAS accumulation in different wild boar tissues and, consequently, affect their interpretation for biomonitoring and human exposure assessment. To the best of our knowledge, this is the first review specifically addressing the occurrence and implications of PFAS in wild boar while linking these interconnected aspects.

## 2. Literature Search and Review Methodology

This study was conducted as a narrative review based on a structured search of the scientific literature. The literature search was last updated in June 2026 and was performed using the Web of Science Core Collection and PubMed databases. The search strategy used combinations of PFAS-related terms, including “PFAS”, “perfluoroalkyl substances”, “per- and polyfluoroalkyl substances”, “perfluorinated compounds”, “perfluorooctane sulfonate”, “PFOS”, “perfluorooctanoic acid”, and “PFOA”, with terms related to wildlife and game, including “wild animals”, “game”, “game meat”, “game species”, “deer”, and “wild boar”.

Study selection followed predefined criteria. Inclusion criteria were peer-reviewed studies published primarily between 2021 and 2026 that evaluated the occurrence, distribution, toxicokinetics, bioaccumulation, environmental monitoring, or potential human exposure to PFAS in wild boar (*Sus scrofa*) or other wildlife species. Exclusion criteria were non-English publications, articles without full-text access, studies lacking a clearly described methodology used, and studies that did not provide extractable data in any format (including their tables or supplementary materials). Additionally, studies focusing exclusively on laboratory animals or domestic livestock without relevance to wildlife or game meat exposure were excluded.

Retrieved records were initially screened by title and abstract for relevance. Full-text articles meeting the inclusion criteria were subsequently evaluated to synthesize the available evidence. Finally, reference lists of identified publications were manually screened to identify additional relevant studies not captured by the primary database search.

## 3. Toxicokinetics and Tissue Distribution of PFAS

Animal organisms may be exposed to PFAS through ingestion, inhalation, and dermal absorption, with ingestion currently considered the primary route of exposure in wildlife. Once inside the organism, most of these compounds exhibit minimal metabolic transformation and remain chemically stable [34,35].

However, certain precursor PFAS can be biotransformed in vivo, resulting in the formation of more stable perfluoroalkyl acids (PFAAs) [36,37,38]. Following absorption, PFAS are partly eliminated via urinary and faecal excretion, while the remainder is distributed across internal compartments [34,39,40]. The biological half-life of PFAS is strongly influenced by the physicochemical properties of individual compounds. In general, shorter-chain PFAS exhibit higher water solubility and lower protein-binding affinity, resulting in faster elimination, while longer-chain compounds show stronger binding and increased retention. Sulfonates also typically display longer biological half-lives than carboxylates (e.g., perfluorobutanoic acid (PFBA) < perfluorobutanesulfonic acid (PFBS) < PFOA < PFOS) [34,41].

Pronounced interspecies differences in PFAS elimination kinetics have been reported. For instance, PFOA exhibits half-lives ranging from a few hours in rabbits to several weeks in birds, and extending to years in humans and pigs, highlighting strong species-dependent variability in PFAS toxicokinetics [34,42]. Similar variability has also been observed for PFOS, with reported half-lives approximately 5 years in humans and deer liver, compared to over 10 years in some marine wildlife species [21]. Data on PFAS elimination half-lives in wild boar remain scarce, making it difficult to characterize species-specific elimination kinetics in this species.

Consequently, factors that may contribute to interindividual variability in PFAS concentrations, including sex and age, warrant consideration. Mertens et al. (2025) reported higher PFAS concentrations in male wild boar and suggested that this pattern may be related to mechanisms described in other species, including maternal transfer during gestation and lactation and partial elimination by females [43]. However, other studies conducted in wild boar have not identified statistically significant differences in PFAS concentrations between sexes [31,44,45], indicating that sex-related differences may not be consistent across studies and may depend on the specific study context. Age-related patterns have likewise been reported by Mertens et al. (2025), who observed elevated PFAS concentrations in young individuals during the lactation period, followed by a decline associated with rapid body growth, and a subsequent increase in older animals, consistent with progressive bioaccumulation over time [43]. In contrast, several studies in wild boar have not observed statistically significant associations between age and PFAS concentrations [31,33,44,46]. These apparently inconsistent findings suggest that the contribution of sex and age to PFAS accumulation in wild boar may be influenced by differences in exposure conditions, individual physiology, tissue distribution, and the specific PFAS compounds investigated.

Protein-rich tissues, including the liver, kidneys, muscle, and blood serum, represent the primary sites of accumulation for these compounds [30,47]. Unlike lipophilic contaminants such as polycyclic aromatic hydrocarbons (PAHs) and polychlorinated biphenyls (PCBs), PFAS do not preferentially accumulate in adipose tissue, which is largely attributed to their strong affinity for proteins, particularly serum albumin [48]. In wild boar, the liver appears to be the principal site of PFAS accumulation, with consistently higher concentrations reported than in kidney and muscle tissues. For example, Schröder et al. (2024) observed approximately four-fold higher concentrations of C9–C14 perfluorocarboxylic acids (PFCAs) and PFOS in wild boar liver compared with kidneys, while Tavoloni et al. (2023) reported up to 28-fold higher total PFAS levels in liver than in muscle [33,46].

The mechanisms underlying this tissue distribution are likely compound- and tissue-dependent. Evidence from experimental and human studies indicates that PFAS can interact with serum proteins, including albumin, and with tissue-specific proteins and phospholipids [48,49,50]. Transporter-mediated processes, including organic anion transporters, have also been implicated in PFAS hepatic and renal disposition in humans and laboratory animals [51,52].

However, the extent to which these mechanisms contribute to PFAS disposition in wild boar remains to be established. A recent multi-tissue study analysing plasma, liver, kidney, lung, spleen, heart, and skeletal muscle provided more comprehensive insight into PFAS distribution in this species [43]. The highest median concentrations for the sum of PFOS, PFOA, PFHxS and PFNA (Σ4PFAS) were reported in liver, followed by kidney, plasma, and lung, whereas substantially lower levels were observed in spleen, heart, and skeletal muscle [43]. In lung, spleen, skeletal muscle, and heart, the accumulation of PFCAs (C8–C13) increased with increasing carbon-chain length, a pattern consistent with passive distribution driven by physicochemical properties such as hydrophobicity [43]. In contrast, liver and kidney exhibited more variable distribution coefficients and higher accumulation factors, suggesting an additional contribution of chain-length-dependent active transport processes [43]. The strongest accumulation was observed for PFOS in liver tissue, whereas shorter-chain PFAS, including PFHxA and PFBS, showed little or no tissue accumulation and were predominantly detected in plasma [43]. Collectively, these findings indicate that PFAS distribution in wild boar varies according to both tissue type and PFAS chain length, with protein binding and passive physicochemical partitioning likely contributing to distribution, while the relative contribution of active transport and tissue-specific binding mechanisms remains incompletely characterized in this species.

To accurately interpret wild boar tissue data in biomonitoring, these specific toxicokinetics must be carefully integrated. By analogy with other mammalian species, long-chain PFAS are hypothesized to bind strongly to serum proteins and renal organic anion transporters (OATs), which would significantly delay their excretion [51,52]. If operational in wild boar, such slow elimination kinetics would mean that tissue residues primary reflect long-term, chronic background exposure rather than recent, short-term environmental fluctuations. Furthermore, the non-uniform distribution coefficients among internal organs directly affect data evaluation. Mechanisms such as active transport and tissue-specific protein binding are thought to drive high hepatic and renal tropism, creating a sharp contrast between target organs and skeletal muscle. Consequently, monitoring programs relying solely on muscle tissue (game meat) will severely underestimate regional terrestrial pollution, whereas the liver offers valuable data for identifying environmental hot-spots and cumulative exposure. Because the most suitable matrix depends on the specific monitoring objective—whether assessing recent exposure via blood plasma, long-term trends via liver, or dietary intake via muscle—researchers must account for these tissue-specific partitioning factors to avoid misinterpreting biological variations as environmental trends.

Similar liver-dominated accumulation patterns have also been reported in other wildlife species. In grey seals (*Halichoerus grypus*) and otters (*Lutra lutra*), liver concentrations exceeded those in muscle by approximately 5-fold and up to 28-fold, respectively, with PFOS identified as the dominant compound in both tissues [44]. Besides differences in absolute concentrations, the authors also evaluated relative PFAS composition and observed that short-chain PFCAs (nC < 7) and precursor compounds contributed proportionally more to muscle tissue than to liver [44]. These findings suggest that, although liver preferentially accumulates persistent long-chain PFAS, other tissues may contain relatively higher proportions of more mobile compounds. Comparable compound-specific distribution patterns have also been described in roe deer (*Capreolus capreolus*), where PFOS, PFOA, and PFBS were predominantly associated with liver tissue, while PFBA and PFHxS were more frequently detected in plasma [53]. This pattern generally aligns with the higher mobility of short-chain compounds like PFBA in circulation, whereas persistent long-chain PFAS (PFOS and PFOA) accumulate in the liver. However, the detection of PFBS (short-chain PFSA) in liver and PFHxS (long-chain PFSA) in plasma indicates that tissue distribution is influenced not only by chain length, but also by functional group and species-specific binding affinities.

Taken together, available evidence indicates that PFAS distribution in wild boar is strongly compound-, tissue-, and potentially species-dependent. Long-chain PFAS, particularly PFOS, show a greater tendency to accumulate in liver and, to a lesser extent, kidney, whereas shorter-chain PFAS are more frequently detected in plasma and exhibit lower tissue accumulation [43]. These patterns are consistent with the combined influence of physicochemical properties, protein binding, tissue-specific interactions, and potentially transporter-mediated processes. However, evidence for protein binding and transporter-mediated mechanisms derives predominantly from human and experimental studies [43,44,45,46,47], whereas evidence from wild boar remains limited and largely inferential. The multi-tissue findings reported by Mertens et al. (2025) [43] provide evidence that tissue distribution in wild boar may involve mechanisms beyond passive physicochemical partitioning, particularly in liver and kidney, but the specific molecular pathways responsible for these patterns remain to be established. This distinction is important for interpreting tissue concentrations in wild boar and for selecting appropriate matrices for PFAS biomonitoring.

## 4. PFAS in Wildlife: Differences Between Species

The occurrence of PFAS in wild game species remains relatively underexplored compared with commercial livestock and widely consumed animal products, despite its relevance for environmental assessment and food safety. Although interest in this area has increased in recent years, available data are still limited and unevenly distributed across species and regions [11,34,54]. The bioaccumulation and tissue distribution of PFAS in wildlife are highly compound-specific, being strongly influenced by chain length, functional groups, trophic position, and feeding ecology. While long-chain PFAS consistently exhibit increasing concentrations from herbivores to omnivores and carnivores across ecosystems, short-chain homologs generally show limited biomagnification potential due to faster elimination kinetics [44,53,55]. Several studies have further confirmed significant trophic magnification of PFOS, which consistently increases across trophic levels and results in elevated concentrations in apex predators [56,57,58,59].

Herbivorous species generally show lower total PFAS concentrations than omnivorous and carnivorous species, reflecting their lower trophic position and reduced potential for biomagnification of long-chain compounds. As trophic transfer within food webs is primarily driven by dietary exposure to bioaccumulative homologs, organisms feeding predominantly on plant material receive lower contaminant inputs compared to higher trophic level species relying on animal-based food sources [60,61]. In addition to total concentrations, trophic level also influences PFAS composition profiles. Herbivorous species are frequently characterized by a predominance of short-chain PFASs, such as PFBS, whereas higher trophic level organisms tend to accumulate long-chain PFAS, including PFOS and long-chain PFCA [32,44,62].

Nevertheless, interspecific variability exists even within trophic groups, indicating that physiological and ecological traits, as well as cumulative exposure, may further influence PFAS accumulation patterns [53,60]. In addition, environmental and habitat-related factors contribute to observed differences, as PFAS exposure patterns are shaped by variation in contamination sources and environmental transport pathways across terrestrial, freshwater, and (semi-)aquatic ecosystems [44].

**Figure 1:**
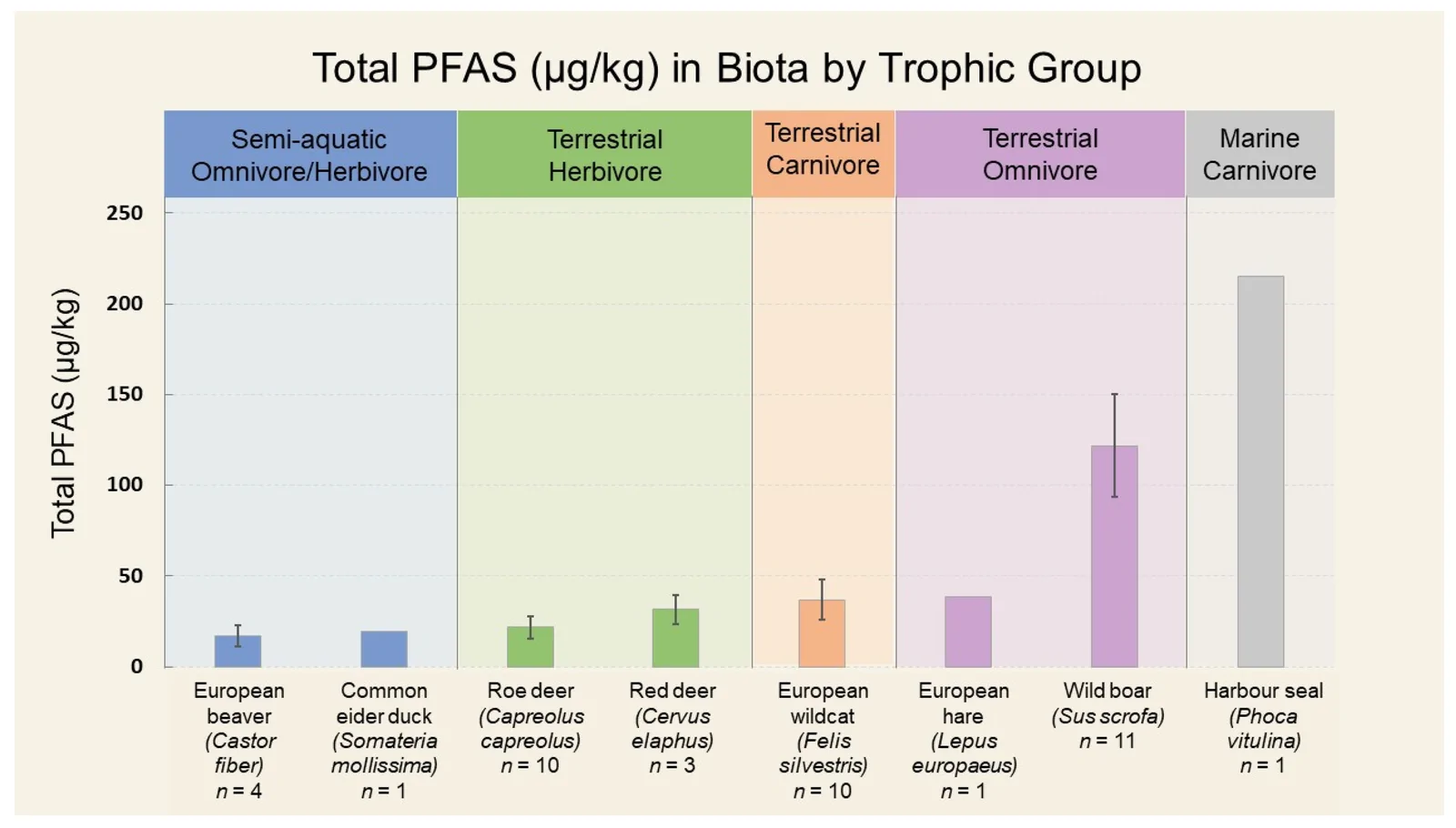
Comparative overview of per- and polyfluoroalkyl substances (PFAS) concentrations in liver of wildlife species according to trophic level and habitat type (data adapted from Guckert et al. (2023) [44]). Bars represent mean concentrations (*n* > 1) or individual values (*n* = 1), with sample sizes (*n*) and error bars indicating standard deviation (SD) where applicable.

This interaction between trophic position and habitat type was illustrated by Guckert et al. (2023) [44], who reported a clear ecological gradient in PFAS contamination across wildlife species (Figure 1). However, when evaluating these contamination profiles, terrestrial and aquatic/semi-aquatic food webs must be considered separately, as exposure pathways and accumulation mechanisms differ between ecosystems. In aquatic and semi-aquatic ecosystems, continuous direct exposure to contaminated water and sediments, combined with aquatic food web transfer, drives substantially elevated accumulation in apex predators such as harbour seals (*Phoca vitulina*). Consequently, higher PFAS concentrations in aquatic species should not be interpreted solely as a consequence of a higher trophic position, but rather as a reflection of habitat-specific matrix exposure. Within terrestrial ecosystems specifically, where exposure is primarily driven by dietary intake and soil ingestion, a distinct trophic gradient is observed. Omnivorous species, particularly wild boar, exhibited the highest mean total PFAS concentrations, followed by European wildcat (*Felis silvestris*) and hare (*Lepus europaeus*), with lower levels observed in red deer (*Cervus elaphus*), chamois (*Rupicapra rupicapra*), and roe deer (*Capreolus capreolus*) [44]. Within these compiled profiles, PFOS consistently emerged as the dominant compound, accounting for more than half of the total PFAS burden across most species, followed by long-chain PFCAs. Furthermore, higher trophic level groups, such as carnivores and omnivores, exhibited much wider individual variations in total concentrations compared to the more uniform profiles found in herbivores [44]. These findings demonstrate that PFAS accumulation in wildlife is jointly governed by trophic ecology, compound-specific properties, and habitat-associated exposure pathways, resulting in pronounced variations between ecological compartments.

## 5. Wild Boar as a Bioindicator of PFAS Contamination

Wild boars exhibit ecological, physiological, and behavioral traits that support their potential as bioindicators of environmental contamination, particularly in terrestrial ecosystems. Their opportunistic omnivorous diet, which heavily involves rooting through topsoil to consume plant roots, invertebrates, and small vertebrates, results in direct and continuous exposure to soil-bound contaminants. Additionally, their relatively long lifespan and position as apex opportunistic feeders allow for significant bioaccumulation over time [30,63].

Table 1 summarizes the key studies reporting PFAS concentrations in wild boar, including information on the biological matrix, study location, sampling year, sample size and sex distribution, analyzed PFAS compounds, analytical methods, reported concentrations (µg/kg), detection/quantification limits (LOD/LOQ) and the main findings.

When comparing these findings across different studies, several methodological and reporting differences must be considered. First, some studies express PFAS concentrations on a dry weight basis, which naturally results in higher absolute values than those reported on a fresh weight basis. Second, analytical sensitivity differs among laboratories, with limits of quantification (LOQ) and detection (LOD) varying depending on the specific instrumental setup and target list of analytes. Third, tissue selection strongly influences reported concentrations; liver consistently yields higher PFAS burdens than muscle or other edible tissues, reflecting the organ-specific accumulation patterns of these substances. Finally, demographic variables such as age and sex are inconsistently reported or adjusted for, despite their potential influence on exposure duration and metabolic clearance.

From a landscape-level perspective, comparative studies suggest that wild boars may serve as more sensitive terrestrial bioindicators than domesticated animals. Mikolajczyk et al. (2024) [32] reported consistently higher PFAS concentrations in wild boar compared to livestock, with a decreasing trend observed from wild boar to cattle, followed by pigs and poultry, when considering key compounds such as PFOS, PFOA, PFNA, and PFHxS. Linear PFOS was detected in all wild boar samples, whereas its occurrence was substantially lower in cattle (33%) and pigs (3%). Moreover, mean PFOS concentrations in pig and cattle muscle tissue were approximately one order of magnitude lower than those observed in wild boar. These differences reflect the controlled feeding conditions and limited environmental exposure of farm animals, in contrast to the continuous and heterogeneous exposure experienced by wild boars [32].

Building on this bioindicative potential, several studies have linked wild boar PFAS burdens directly to environmental matrices, while others demonstrate regional spatial trends even without parallel soil or water sampling.

Rupp et al. (2023) suggested a strong relationship between PFAS concentrations in soil and their accumulation in wild boar liver, indicating that wild boar liver may reflect environmental contamination levels in specific local settings [30]. Importantly, PFAS patterns in wild boar liver appeared to mirror those observed in soil, particularly for PFCAs, while concentrations in liver were substantially higher—often by one to two orders of magnitude—indicating significant bioaccumulation potential. In their study, PFAS levels and profiles clearly differed across areas associated with industrial activity, contaminated paper sludge, and background contamination conditions. The highest total PFAS levels were observed in the industrial area (∑PFAS 944 μg/kg), followed by the sludge-associated area (567 μg/kg), while substantially lower levels were detected in the background area (124 μg/kg). In addition, compound-specific patterns reflected dominant emission sources, with PFOS prevailing in the sludge-impacted area, whereas PFOA was the predominant compound in the industrial zone. The detection of emerging PFAS such as 4,8-dioxa-3H-perfluorononanoic acid (DONA) and hexafluoropropylene oxide dimer acid (HFPO-DA), used as substitutes for legacy compounds, further supports the responsiveness of wild boar to contemporary contamination inputs, although factors such as animal movement, diet, and metabolic variations must be considered when interpreting these patterns. Overall, these findings suggest that wild boar may not only reflect contamination intensity, but also provide insights into its potential origin and composition [30].

Subsequent studies have further explored the use of wild boar as a bioindicator of PFAS contamination, although often without parallel analysis of environmental matrices such as soil. Instead, these investigations relied primarily on PFAS concentrations in wild boar tissues to assess spatial patterns of contamination and their relationship with anthropogenic pressures.

These studies collectively point to the potential applicability of wild boar in biomonitoring frameworks. Felder et al. (2026) [31] analyzed PFAS concentrations in wild boar liver samples collected from 18 hunting zones across Germany and observed a proportional increase in PFAS levels with the extent of urban and/or industrial land use within a defined radius of each zone. More detailed land-use analyses revealed compound-specific associations, with elevated concentrations of PFOA, PFOS, PFNA, and PFDA linked to areas with higher degrees of urbanization, while PFUnDA showed a stronger association with agricultural land use. This differentiation suggests that distinct emission sources and pathways may be reflected in the PFAS profiles observed in wild boar tissues. In this context, the integration of land-use data with precise information on the origin of sampled individuals was highlighted as a particularly valuable approach, as it may facilitate the identification of both intentional and unintentional PFAS releases, including those associated with agricultural practices [31]. Similar spatial trends were also observed in the study by Rupp et al. (2023), where elevated concentrations were consistently associated with areas of known industrial impact [30].

However, not all observed contamination patterns can be explained solely by direct local sources. Evidence from studies conducted in remote and protected environments indicates that wild boar can also reflect long-range environmental transport processes. In such areas, where direct anthropogenic inputs are minimal, detectable PFAS concentrations have nonetheless been reported in wild boar tissues, suggesting atmospheric deposition as a relevant exposure pathway [46].

A notable example is provided by Schröder et al. (2024) [46] who investigated PFAS levels in wild boar tissues collected within a protected national park, where all river systems originate within the park boundaries and human activity is strictly limited. As a result, external inputs via surface water and direct anthropogenic influences are minimal, and PFAS deposition is assumed to occur primarily through atmospheric transport [46]. Despite the absence of immediate contamination sources, detectable PFAS concentrations were found in both liver and kidney samples, indicating that even relatively remote ecosystems are not exempt from PFAS exposure. Interestingly, the study observed that PFAS concentrations generally decreased with increasing carbon chain length. This pattern is consistent with the hypothesis that atmospheric transport and deposition may contribute to PFAS exposure in this remote environment. However, this relationship should be interpreted with caution, as the observed chain-length pattern may also reflect compound-specific differences in environmental mobility, bioaccumulation, protein binding, tissue distribution, and toxicokinetics. However, an exception was noted for perfluorotridecanoic acid (PFTrDA), which showed higher concentrations than expected, suggesting the influence of a more localized or specific emission source. In this context, the authors identified a potential contributor in the form of a PFAS manufacturing facility located approximately 120–140 km from the study area, indicating that industrial emissions can impact wildlife even at considerable distances [46].

Together, these findings are consistent with the view that wild boar can capture both diffuse long-range atmospheric inputs and localized emission sources, even in environments with minimal direct anthropogenic influence. While tissue concentrations are inherently influenced by animal mobility, individual foraging behavior, and geographical boundaries, these studies collectively support the utility of wild boar as a valuable bioindicator for assessing exposure trends and fate processes in terrestrial ecosystems.

## 6. Implications for Human Exposure Through Wild Boar Consumption

The European Food Safety Authority (EFSA) has identified dietary intake as the primary route of human exposure to PFAS [13]. In response to growing toxicological evidence, EFSA established in 2020 a group tolerable weekly intake (TWI) of 4.4 ng/kg body weight for the sum of four major PFAS compounds—PFOS, PFOA, PFNA, and PFHxS—thereby providing a benchmark for risk assessment of dietary exposure [64].

At the regulatory level, Commission Regulation (EU) 2023/915 defines maximum levels for these compounds in various food categories. For meat of game animals (excluding bear meat), maximum levels are set at 5.0, 3.5, 1.5, and 0.6 μg/kg for PFOS, PFOA, PFNA, and PFHxS, respectively, with a maximum level of 9.0 μg/kg established for the sum of these compounds. Substantially higher maximum levels are set for offal of game animals (with the exception of bear offal), at 50, 25, 45, and 3.0 μg/kg for PFOS, PFOA, PFNA, and PFHxS, with their sum limited to 50 μg/kg, reflecting the known accumulation of PFAS in metabolically active organs such as the liver [23].

Outside Europe, regulatory approaches specifically addressing PFAS in wild game meat are limited, with most frameworks focusing on environmental monitoring or general wildlife management guidance rather than establishing specific maximum levels for game species. Consequently, the European Union remains the only region with harmonised, legally binding maximum levels for PFAS in game meat and offal [65,66].

Game meat has long played a significant role in human nutrition, while hunting remains an important activity, deeply embedded in cultural traditions and heritage. Beyond its nutritional function, hunting is also recognised as a recreational and socio-cultural practice, as well as a tool for wildlife management, with sustainability increasingly incorporated into modern hunting practices [67]. A wide range of wild game species are consumed, including deer species, wild boar, pheasant, partridge, hare, grouse, and wild waterfowl. Among these, wild boar and red deer represent the most important large game species, particularly in Central and Southern Europe, due to their wide distribution and high abundance [67,68]. Wild boar meat is also considered nutritionally valuable due to its high content of omega-3 polyunsaturated fatty acids and essential minerals, particularly iron, zinc, and phosphorus [69]. Recent decades have seen a marked increase in wild game populations across Europe, especially wild boar, driven by changes in land use, forest expansion, rural depopulation, reduced hunting pressure in some regions, and wildlife management practices. This population growth has increased the availability of game meat but has also raised concerns regarding human–wildlife interactions and disease risks [67].

Despite this increased availability, game meat consumption remains relatively low, highly unevenly distributed, and typically episodic. For instance, annual per capita consumption in Germany has been estimated at approximately 200–400 g. Similar trends were reported in Italy (100–300 g annual per capita), illustrating the generally marginal contribution of game meat to overall dietary intake [30,70].

Higher intake frequencies are predominantly observed among hunters and their families, reflecting the strong link between consumption patterns, hunting practices, and cultural context across Europe [30,68]. Consequently, hunters and their families may experience higher dietary exposure to PFAS due to their more frequent consumption of wild game, particularly offal. While consumption of wild boar liver is negligible in the general population, it is more regularly included in the diet of hunters, with a reported median intake of 13 g/week in Italy [30]. This distinction is particularly relevant given the pronounced differences in PFAS accumulation between tissue types, with substantially higher concentrations observed in offal compared to muscle. Similar findings were reported by Draghi et al. (2025), who confirmed that PFAS concentrations in offal frequently exceed EU maximum levels, further highlighting the relevance of these tissues for dietary exposure assessment and targeted monitoring strategies [48].

Reported PFAS exposure estimates associated with the consumption of wild boar muscle and offal, including differences according to tissue type, consumption frequency, and age group, are summarized in Table 2.

When evaluating these dietary risk assessments, however, a simple comparison of final exposure values can be misleading without considering the underlying assumptions made in each study. As detailed in Table 2, different authors utilize widely varying scenarios for dietary intake and consumption frequencies. For example, high-exposure models for hunters range from a steady weekly intake of 13 g in Italian cohorts [30] to a strict calculated maximum threshold of 0.75 g/week in German risk assessments [45], while other models assume single large annual portions. Additionally, the baseline body weights and age groups used to evaluate vulnerable populations, such as children, often differ across regions. Because these baseline calculation parameters and dietary habits are not standardized, the resulting percentages of the EFSA TWI should be interpreted as regional examples of potential risk rather than direct, equivalent matches.

Risk assessment studies consistently identify liver consumption as the most critical exposure pathway, whereas muscle tissue plays a secondary role. Rupp et al. (2023) [30] showed that consumption of a single 200 g portion of wild boar liver per year may be sufficient to exceed the EFSA tolerable weekly intake, primarily due to elevated PFOS concentrations. Under regular consumption scenarios, exposure levels may remain persistently above health-based guidance values, even when sourcing from relatively low- contaminated hunting areas [30]. Similarly, Tavoloni et al. (2023) reported that exposure from wild boar muscle remains well below the TWI, whereas liver consumption may result in exposures several-fold higher than the threshold value [33]. These findings are further supported by Felder et al. (2022) [45], who estimated that a tolerable intake of wild boar liver may be as low as 0.75 g per week under median contamination scenarios, with even moderate consumption leading to exceedance when combined with background dietary exposure. Reported liver concentrations exceeding 400 μg/kg underline the magnitude of contamination and its relevance for food safety considerations [45].

Conversely, while muscle tissue generally accumulates lower PFAS levels, it remains a notable and widespread contributor to dietary intake. Mikolajczyk et al. (2024) [32] analysed PFAS contamination in muscle tissue from wild boar, cattle, deer, pigs, and poultry and found that, although none of the samples exceeded the regulatory limits established by Commission Regulation (EU) 2023/915 [23], wild boar meat represented a disproportionately large contributor to dietary PFAS exposure. Under frequent consumption scenarios, consumption of wild boar meat accounted for up to 63% of the TWI in children and 21% in adults, whereas exposure associated with poultry, pork, beef, and venison remained below 1% of the TWI [32]. The higher contribution observed in children is primarily related to exposure relative to body weight. Pregnancy and childhood may also warrant particular attention because they represent sensitive periods of development. However, these considerations should not be interpreted as evidence of an epidemiologically established increased health risk specifically associated with wild game consumption. Rather, they indicate that frequent consumption of wild boar meat may result in a relatively higher dietary PFAS exposure, particularly in children.

While most dietary risk assessments of PFAS in wild game have focused on estimated intake rather than internal exposure, human biomonitoring studies provide evidence of associations between consumption of animal-derived foods and circulating PFAS concentrations. In a large European study of 1957 adolescents from nine countries, serum PFOS concentrations were associated with offal consumption, with offal consumers showing 14% higher PFOS concentrations than non-consumers [71]. More recently, an analysis of National Health and Nutrition Examination Survey (NHANES) data from 2003 to 2020 found that meat intake was modestly associated with serum PFAS concentrations in the US population [72]. In a highly contaminated food exposure setting in Korsør, Denmark, the amount of consumed PFAS-contaminated veal was identified as a major predictor of serum PFOS and PFHxS concentrations, and ingestion of the contaminated meat was associated with a dose–response relationship with serum concentrations [73]. However, evidence specifically linking consumption of wild game meat or offal to PFAS body burden in humans remains limited.

In addition, emerging land-use-based risk assessment approaches have been proposed to identify contamination hotspots by integrating environmental data with information on animal origin, thereby improving source attribution and monitoring efficiency, particularly in industrial and agricultural landscapes [31].

From a risk management perspective, precautionary measures have already been implemented in several European regions. In Germany, food safety authorities recommend avoiding consumption of wild boar liver from specific hunting areas [30], and a formal ban on the sale of wild boar liver was introduced in the federal state of Hesse in 2021 following consistently elevated PFAS levels [45]. Muscle meat remains permitted due to comparatively lower contamination levels and limited consumption frequency, reflecting a risk-based regulatory approach.

Overall, although game meat consumption remains limited in the general population, heterogeneity in consumption patterns, increasing wild boar populations, and the pronounced bioaccumulation of PFAS in offal collectively highlight its relevance as a dietary exposure pathway. Continued monitoring, refined exposure assessment methodologies, and harmonized regulatory approaches are therefore essential to ensure effective risk management, particularly for high-consumption subgroups such as hunters.

## 7. Knowledge Gaps and Future Perspectives

Despite the growing body of evidence on PFAS occurrence in wild boar, several important knowledge gaps remain. Current research is geographically unbalanced, with most studies originating from Germany and Italy, while comparatively fewer investigations have been conducted in other European countries, including Poland and the Czech Republic. Outside Europe, information remains extremely limited despite the wide global distribution of wild boar, particularly in North America, South America, and Asia. This geographical imbalance limits the extrapolation of current findings to other environmental, ecological, and dietary contexts.

Another important limitation concerns the methodological heterogeneity among available studies. Differences in analytical approaches, target PFAS lists, limits of detection and quantification, biological matrices, and the expression of concentrations, particularly fresh weight versus dry weight, complicate direct comparisons among studies and hinder robust assessments of spatial and temporal trends. Greater harmonization of sampling strategies, target compound selection, reporting units, and quality assurance criteria would improve data comparability and facilitate the integration of wild boar into broader biomonitoring frameworks.

Current knowledge is also focused primarily on a relatively small number of legacy PFAS, particularly PFOS, PFOA, PFNA, and PFHxS. Future studies should expand target analyte lists and, at the same time, move beyond the traditional single-compound approach by investigating PFAS as complex mixtures. This is particularly relevant because wildlife and humans are simultaneously exposed to multiple legacy and emerging PFAS, whose combined effects may differ from those of individual substances. Greater emphasis on mixture toxicity and cumulative exposure would provide a more realistic basis for environmental and human health risk assessment and support the development of group-based regulatory strategies.

Although several studies have demonstrated relationships between PFAS concentrations in wild boar tissues and environmental contamination, integrated investigations simultaneously assessing environmental matrices, dietary sources, and animal tissues remain uncommon. Such multidisciplinary approaches would improve the understanding of PFAS transfer pathways within terrestrial food chain, facilitate source attribution, and help identify contamination scenarios of greatest relevance for both biomonitoring and food safety. From a toxicokinetic perspective, the mechanisms governing tissue-specific PFAS accumulation in wild boar require further clarification. While protein binding, hydrophobicity-related partitioning, and transporter-mediated processes are likely to contribute to PFAS distribution, their relative importance across compounds and tissues remains insufficiently characterized in this species. Future research should therefore clarify the role of plasma protein binding, membrane transporters, renal elimination, and other species-specific physiological processes influencing PFAS retention and elimination.

Finally, further refinement of human exposure assessments is warranted. Existing studies mainly evaluate dietary exposure using average consumption scenarios, whereas cumulative exposure from multiple dietary sources, regional dietary habits, and mixtures of legacy and emerging PFAS remains poorly characterized. Greater attention to high-consumption subgroups, particularly hunters and their families, and the integration of environmental monitoring, wildlife biomonitoring, and dietary exposure assessment within a One Health framework would provide a stronger basis for future risk assessment and regulatory decision-making.

In summary, several major limitations affect the overall interpretation and generalizability of the current evidence on PFAS in wild boar. Geographically, research remains concentrated in a few European countries, limiting the extrapolation of findings to broader global contexts. Methodologically, substantial heterogeneity exists across studies, including differences in target PFAS lists, analytical sensitivity (LOD/LOQ), biological matrices sampled, and data reporting units—specifically the use of wet- versus dry-weight concentrations, which complicates direct numerical comparisons. Furthermore, biological and ecological variables such as age, sex, seasonal dietary shifts, and animal movement are inconsistently accounted for or reported across the literature, despite their potential to influence exposure dynamics and tissue residues. Together, these factors limit direct quantitative comparisons across the literature and restrict the generalizability of region-specific conclusions.

## 8. Conclusions

Wild boar (*Sus scrofa*) has been demonstrated as a valuable sentinel species for environmental PFAS contamination, showing clear relationships between PFAS concentrations in tissues and levels detected in surrounding environmental matrices such as soil. However, data interpretation must account for compound-specific toxicokinetics, including delayed excretion that may be driven by strong protein-binding and transporter affinities, which likely cause tissue residues to reflect chronic, long-term exposure rather than recent environmental fluctuations. Liver and kidney have been identified as key accumulation organs, where PFAS concentrations appear to be driven by both passive partitioning linked to hydrophobicity and proposed active transport mechanisms, suggesting substantially higher internal burdens compared to skeletal muscle. Consequently, biomonitoring frameworks relying solely on muscle tissue may underestimate regional pollution. While the liver appears to be a particularly informative matrix for assessing cumulative PFAS burden and identifying potential contamination hotspots, the suitability of a specific matrix depends on the biomonitoring objective; in particular, muscle tissue remains directly relevant to dietary exposure assessment. Given the current geographical focus primarily on a few European countries, as well as confounding factors such as animal movement and seasonal diet changes, the wild boar should be interpreted as a highly relevant but complex bioindicator rather than an absolute baseline for local contamination.

Beyond its environmental relevance, PFAS contamination in wild boar is also important from a human health perspective. Hunters and their families represent the most exposed population group due to higher and more frequent consumption of wild game, particularly offal. Current research is still largely concentrated in a limited number of European countries, where precautionary recommendations and consumption advisories have already been issued in areas with elevated contamination levels.

Comparable tissue-specific distribution patterns appear to occur in other wildlife species, such as roe deer (*Capreolus capreolus*), where liver tends to accumulate persistent PFAS, while short-chain compounds are more frequently found in plasma, suggesting similar mechanisms of compound-specific partitioning across species.

Overall, expanding PFAS monitoring in wild boar and other game species, both within Europe and globally, is essential for improving environmental surveillance and supporting human and wildlife risk assessment. Future studies should therefore also include other commonly consumed game species, including deer, roe deer, pheasants, and wild ducks, in order to better understand PFAS transfer through terrestrial food webs and broader ecosystem contamination patterns.

## Figures and Tables

**Table 1:** Overview of studies reporting PFAS concentrations in wild boar.

Matrix	Study & Sample Details	Analytes, Method & LOQ/LOD (µg/kg)	Reported Concentrations (μg/kg)	Key Findings & Reference
Liver	Brandenburg, Germany (2019–2022) *n* = 164 (M/F)	16 PFAS (HPLC-MS/MS) LOQ = 0.2 LOD = 0.07	PFOS: median 235 PFNA: median 15 PFDA: median 9.7 PFOA: median 5.3	Long-chain PFCAs and PFSAs were detected in >90% of all samples, with PFOS concentrations being at least 10-fold higher compared to other PFAS. Local habitat characteristics influenced PFAS levels and patterns in wild boar livers. Values were expressed on a dry weight basis (2–3 times higher than wet weight) [31].
Liver and muscle	Northern Italy year NR *n* = 20 (sex NR)	23 PFAS (LC-MS/MS) LOQ = 0.03 LOD = 0.01	15 PFAS mean: 0.440–424.37 (liver) 15 PFAS mean: 0.284–42.65 (muscle)	All analyzed PFAS were detected in 100% of samples. PFOA was the most abundant compound in liver, whereas PFHxS dominated in muscle. PFOS levels remained below the LOQ in both tissues [48].
Plasma and various tissues	Germany (2021–2024) *n* = 82 (M/F)	11 PFAS (UPLC-MS/MS) LOD/LOQ = NR	Σ4 PFAS median: 90.2 liver; 9.45 kidney; 7.63 plasma; 6.84 lung; 2.60 heart; 2.46 spleen; 1.03 skeletal muscle	PFOS, PFOA, PFNA, PFHxS were found in all analysed sample. PFOS showed the highest accumulation in the liver, while PFOA, PFBS, PFHxS, PFHpA and PFHxA did not accumulate in any tissue [43].
Liver and kidney	Czech Republic (2017–2018) *n* = 30 (M/F)	30 PFAS (HPLC-MS/MS) LOQ = NR LOD = 0.75–8.9	PFHpA: median 4.1; PFOA: median 75; PFOS: median 170; PFNA: median 230 (liver) PFHpA: median 4.1; PFOA: median 51; PFOS: median 12 (kidney)	Liver showed a 4-fold higher sum of PFAS than kidneys, with PFNA being the most abundant compound and PFOS contributing 30% (no short-chain PFAS detected). Kidney analysis provided no additional profile information. Values were expressed on a dry weight basis [46].
Muscle	Poland (2022–2023) *n* = 14 (sex NR)	13 PFAS (LC-MS/MS) LOQ = 0.001–0.014 LOD = NR	Lower bound Σ13 PFAS: mean 0.93 L-PFOS: max. 1.87	PFOS, PFOA, and PFHxS were detected in all samples, with PFOS dominating the profile (up to 69% of total PFASs across samples). Short-chain PFASs accounted for <10% of total contamination [32].
Liver and muscle	Italy (2019–2020) *n* = 26 (M/F)	19 PFAS (LC-MS/MS) LOQ = 0.010 (0.200 for PFBA) LOD = NR	Liver Σ19 PFAS: mean 87.7 (range 31.9–228) Muscle Σ19 PFAS: mean 3.08 (range 0.59–8.4)	PFOS dominated all liver samples, accounting for over 50% of total contamination. In muscle, PFTrDA was most abundant (25–30%), followed by PFOS (16–17%). Long-chain PFCAs were quantified in all samples, whereas short-chain PFCAs were detected only in wild boars from Umbria [33].
Liver	Germany (2019–2020) *n* = 50 (M/F)	66 PFAS (LC-MS/MS, IC-QTOF) LOQ = 0.010–5 LOD = NR	Σ31 PFAS PS: 567 (PFOS: 426) Σ31 PFAS IE: 944 (PFOA: 650) Σ31 PFAS BC: 124 (PFOS: 82)	PFOS was the dominant compound in the PS and BC areas, while PFOA predominated in the IE area. PFAS patterns in liver closely reflected those detected in soils from the BC and PS areas [30].
Liver	Germany (2015–2020) *n* = 11 (M/F)	66 PFAS (LC-MS/MS, IC-QTOF) LOQ = 0.010–5 LOD = NR	Σ66 PFAS: mean 121.6	PFOS was the dominant PFAS (82 μg/kg), followed by the PFCAs TFA and PFNA (both 11 μg/kg) [44].
Liver and muscle	Germany (2019–2020) *n* = 90 (M/F)	13 PFAS (HPLC–MS/ MS) LOQ = 0.008–0.314 LOD = 0.002–0.094	Liver Σ13PFAS: mean 493 PFOS: mean 117 Muscle PFOS: mean 8.1	In liver samples, PFOS was identified as the dominant compound, accounting for approximately 86% of the total PFAA contamination across the studied animal population. The proportion of short-chain PFAAs, particularly PFCAs, showed strong variability. In muscle, only PFOS was quantitatively detected, with a detection frequency of 95% [45].

Abbreviations: perfluorooctanoic acid (PFOA); perfluorooctane sulfonate (PFOS); perfluorononanoic acid (PFNA); perfluorodecanoic acid (PFDA); perfluorohexane sulfonate (PFHxS); perfluorobutanesulfonic acid (PFBS); perfluoroheptanoic acid (PFHpA); perfluorohexanoic acid (PFHxA); perfluorotridecanoic acid (PFTrDA); trifluoroacetic acid (TFA); sum of PFAS (ΣPFAS); perfluoroalkyl acids (PFAA); perfluorocarboxylic acids (PFCA); areas associated with contaminated paper sludges distributed on arable land (PS); areas associated with industrial emissions of PFAS (IE); areas associated with background contamination (BC); limit of quantification (LOQ); limit of detection (LOD); male and female (M/F); information not reported (NR); high-performance liquid chromatography–tandem mass spectrometry (HPLC-MS/MS); liquid chromatography–tandem mass spectrometry (LC-MS/MS); ultra-performance liquid chromatography–tandem mass spectrometry (UPLC-MS/MS); ion chromatography–quadrupole time-of-flight mass spectrometry (IC-QTOF).

**Table 2:** Reported PFAS exposure estimates associated with the consumption of wild boar muscle and offal.

Tissue	PFAS Included	PFAS Concentration (µg/kg)	Age Group/Body Weight	Amount Consumed	Frequency of Consumption	Calculated Weekly Exposure (ng/kg bw/Week)	Comparation with EFSA TWI (4.4 ng/kg Body Weight)	Reference
Liver	PFOS, PFOA, PFHxS, PFNA	PS 432 IE 792 BC 101	Adult 70 kg	Hunters: 13 g/week General population: 200 g/year	Hunters: weekly General population: annually	Hunters: PS 80, IE 147, BC 19 General population: PS 24, IE 44, BC 5.6	Above EFSA TWI	[30]
		65.3	Adult 70 kg	Hunters: 3–33 g/day	Not reported	30.17	Above EFSA TWI	[33]
		408.59	Adult 70 kg	0.75 g/week (calculated)	Weekly	4.4	Calculated at EFSA TWI	[45]
Muscle	PFOS, PFOA, PFHxS, PFNA	0.95	Adult 70 kg	6–100 g/day	Not reported	0.574	Below EFSA TWI	[33]
	PFOS	7.29	Adult 70 kg	42.25 g/week (calculated)	Weekly	4.4	Calculated at EFSA TWI	[45]
	PFOS, PFOA, PFHxS, PFNA	0.64	Children 23.1 kg Adult 70 kg	100 g/portion (assumed)	Not reported	Children: 2.75; Adults: 0.91	Below EFSA TWI	[32]

Abbreviations: Per- and polyfluoroalkyl substances (PFAS); European Food Safety Authority tolerable weekly intake (EFSA TWI); perfluorooctane sulfonate (PFOS); perfluorooctanoic acid (PFOA); perfluorohexane sulfonate (PFHxS); perfluorononanoic acid (PFNA); areas associated with contaminated paper sludges distributed on arable land (PS); areas associated with industrial emissions of PFAS (IE); areas associated with background contamination (BC).

## Data Availability

No new data were created or analyzed in this study. Data sharing is not applicable to this article.
